# Antioxidant Defense and Ionic Homeostasis Govern Stage-Specific Response of Salinity Stress in Contrasting Rice Varieties

**DOI:** 10.3390/plants13060778

**Published:** 2024-03-09

**Authors:** Vikash Kumar, Ashish K. Srivastava, Deepak Sharma, Shailaja P. Pandey, Manish Pandey, Ayushi Dudwadkar, Harshala J. Parab, Penna Suprasanna, Bikram K. Das

**Affiliations:** 1 Nuclear Agriculture and Biotechnology Division, Bhabha Atomic Research Centre, Mumbai 400085, India; 2 BARC Campus, Homi Bhabha National Institute, Anushaktinagar, Mumbai 400094, India; 3 Department of Genetics and Plant Breeding, Indira Gandhi Krishi Vishwa Vidyalaya, Raipur 492012, India; 4 Analytical Chemistry Division, Bhabha Atomic Research Centre, Mumbai 400085, India

**Keywords:** rice, salt stress, stage-specific salinity, antioxidant defense, ionic homeostasis, salt tolerance

## Abstract

Salt stress is one of the most severe environmental stresses limiting the productivity of crops, including rice. However, there is a lack of information on how salt-stress sensitivity varies across different developmental stages in rice. In view of this, a comparative evaluation of contrasting rice varieties CSR36 (salt tolerant) and Jaya (salt sensitive) was conducted, wherein NaCl stress (50 mM) was independently given either at seedling (S-stage), tillering (T-stage), flowering (F-stage), seed-setting (SS-stage) or throughout plant growth, from seedling till maturity. Except for S-stage, CSR36 exhibited improved NaCl stress tolerance than Jaya, at all other tested stages. Principal component analysis (PCA) revealed that the improved NaCl stress tolerance in CSR36 coincided with enhanced activities/levels of enzymatic/non-enzymatic antioxidants (root ascorbate peroxidase for T- (2.74-fold) and S+T- (2.12-fold) stages and root catalase for F- (5.22-fold), S+T- (2.10-fold) and S+T+F- (2.61-fold) stages) and higher accumulation of osmolytes (shoot proline for F-stage (5.82-fold) and S+T+F- (2.31-fold) stage), indicating better antioxidant capacitance and osmotic adjustment, respectively. In contrast, higher shoot accumulation of Na^+^ (14.25-fold) and consequent increase in Na^+^/K^+^ (14.56-fold), Na^+^/Mg^+2^ (13.09-fold) and Na^+^/Ca^+2^ (8.38-fold) ratio in shoot, were identified as major variables associated with S-stage salinity in Jaya. Higher root Na^+^ and their associated ratio were major deriving force for other stage specific and combined stage salinity in Jaya. In addition, CSR36 exhibited higher levels of Fe^3+^, Mn^2+^ and Co^3+^ and lower Cl^−^ and SO_4_^2−^, suggesting its potential to discriminate essential and non-essential nutrients, which might contribute to NaCl stress tolerance. Taken together, the findings provided the framework for stage-specific salinity responses in rice, which will facilitate crop-improvement programs for specific ecological niches, including coastal regions.

## 1. Introduction

Soil salinization is a global and dynamic problem and is projected to increase in future, under rapidly changing climatic scenarios [1]. In India, over 7 million ha land is covered with saline soil, which is expected to further increase to 11.7 million ha by 2025 [2]. Increased salinity adversely affects the growth and yield of many staple food crops, including rice (*Oryza sativa* L.), which feeds more than half of the world population. Soil salinity levels are measured in terms of electrical conductivity of a saturated soil extract (ECe) which measures the ability of soil water to carry electrical current by virtue of cations (Ca^2+^, Mg^2+^, K^+^, Na^+^, and NH^4+^) and anions (SO_4_^2−^, Cl^−^, NO_3_^−^ and HCO_3_^−^) present in it. Rice is the most salt-sensitive cereal crop, which suffers up to 50% yield losses at ECe 7.2 dS/m [3]. Salt tolerance in rice depends on the genotype, developmental stage and organ of the plant [4]. Therefore, understanding and improving the salt tolerance of rice at different growth stages, will not only lead to the effective use of the saline land, but it will also support sustainable agriculture and alleviation of the world food crisis in future. The effect of salinity stress is manifested sequentially, beginning with osmotic stress buildup, followed by ionic toxicity and subsequently to severe nutritional deficiencies, eventually leading to growth inhibition and yield losses. Salinity stress impairs photosynthetic activity and overall growth, resulting in reduction in plant height, tiller numbers and biomass and induction of partial sterility [5]. The toxic effect of salinity is usually associated with increased accumulation of Na^+^ ions, which disrupt the ionic homeostasis (K^+^/Na^+^) of the plant. Intracellular K^+^ homeostasis is crucial as it regulates the plant’s physiological functions like osmoregulation, chloroplast development, stomatal conductance, cytosolic pH regulation, phloem translocation, and stabilization of membrane potential, ultimately affecting the crop growth and yield [6]. Salinity increases the level of lipid peroxidation, as envisaged by higher malondialdehyde (MDA) levels, resulting in higher membrane damage as a consequence of imbalanced redox management [7].

Plants deploy a two-stage system to counter the adverse consequences of salinity-induced production of reactive oxygen species (ROS). First, enzymatic antioxidants [superoxide dismutase (SOD), catalase (CAT), ascorbate peroxidase (APX), Guiaicol Peroxidase (GPX)] and second, non-enzymatic [ascorbate (ASC), glutathione (GSH), tocopherols, phenolics and flavonoids] antioxidant systems [8]. Akin to ROS homeostasis, ionic homeostasis also plays an important role towards salinity tolerance in plants. Plants are equipped with ion-exclusion or sequestration mechanisms to avoid the toxic build-up of Na^+^ or Cl^−^ in the roots and their subsequent transport to young leaves [9]. Ionic balance is essential to normal functioning and growth of the plant [10]. Nutrient status of plant is negatively affected under salt stress, as higher concentration of Na^+^ and Cl^−^ in soil sap, decreases the uptake of various macronutrients (NO^3−^, PO_4_^3−^, K^+^, Mg^+2^ and Ca^+2^) and micronutrients (Cu^+2^, Mn^+2^, Zn^+2^, Mo^+3^, Fe^+3^ and Co^+3^) essential for growth and development [11]. High Na^+^ and Cl^−^ concentrations in the soil solution may suppress nutrient uptake and result in undesirable ratios of Na^+^: Ca^2+^, Na^+^: K^+^, and Na^+^: Mg^2+^ [12]. This can lead to ionic imbalance, thereby affecting plant’s physiological traits [13]. High salt concentration results in lower N accretion in plants, due to the interaction between Cl^−^ and NO_3_^−^ and Na^+^ and NH_4_^+^, which subsequently reduces plant growth and crop yield [14]. Salinization renders PO_4_^3−^ unavailable to plants due to its precipitation with other cations, such as Ca^2+^, Mg^2+^, and Zn^2+^ depending upon the pH of the soil environment, thereby, inducing salt-induced P deficiency in plants [15]. Although, the salt-stress induced biochemical and physiological changes are well-demonstrated particularly at seedling stage; however, response of salt-stress to different critical stages or multi-stage sensitivity and their associated protection/sensitivity mechanisms have not yet been reported in rice. To fill this gap, the present study was conducted to see whether differential sensitivity to salt-stress similar to coastal saline conditions, exists at different developmental stages in tolerant and sensitive rice varieties and also, to identify the closely related biochemical attributes responsible for protection/sensitive response.

## 2. Material and Methods

### 2.1. Plant Material, Growth Conditions and Stress Treatment

**Figure 1:**
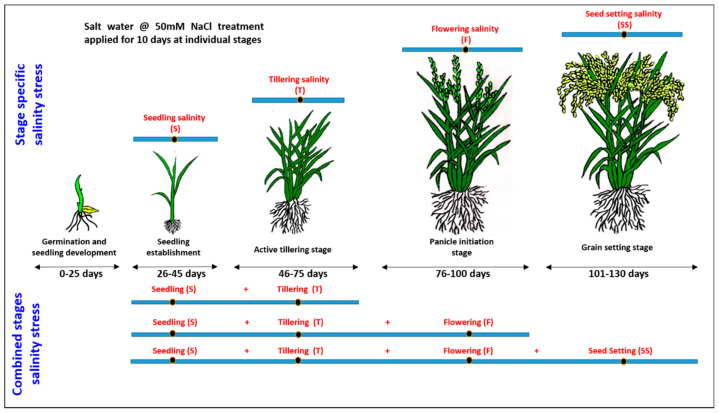
A schematic illustration of the experimental layout.

The present study was conducted at NA&BTD farm of Bhabha Atomic Research Centre, Trombay, Mumbai, India under poly house conditions. The growth conditions were maintained at 30 °C/25 °C day/night temperature, 14/10 h day/night photoperiod, humidity 75 ± 5%, light intensity: 900–1000 µEm^−2^s^−1^. Two rice varieties, namely CSR36 (salt tolerant) and Jaya (salt sensitive), were considered for this study. CSR36 is a commercially grown, widely adapted salt-tolerant (alkaline soil; Ece < 4.0 dSm^−1^, pH > 8.0, dominated by CO_3_^2−^ and HCO_3_^−^ of Na^+^) variety of India, developed through a three-way cross (CSR13 (alkaline soil)/Panvel 2 (coastal saline)/IR36) and possess better grain yield under inland saline conditions [16]. However, its tolerance towards coastal saline conditions is not known. The surface-sterilized seeds of both varieties were germinated for 48 h and hydroponic cultures were established, as per the method described previously [17]. The whole experiment was planted in two treatment sets: (1) stage-specific salinity stress; (2) combined-stages salinity stress (Figure 1). The first set was further divided into 4 treatments namely (i) seedling (S), (ii) tillering (T), (iii) flowering (F) and (iv) seed setting (SS) stage salinity. The second set was divided into 3 treatment sets namely (i) seedling + tillering (S+T), (ii) seeding + tillering + flowering (S+T+F) and (iii) seeding + tillering + flowering + seed setting (S+T+F+SS) stage salinity (Figure 1). Planting was done with three replications/treatment in (Randomized Block Design) RBD and three pots per replication. Four healthy, 30 days old (4-leaf stage), hydroponically grown seedlings of both the varieties were transferred to single plastic pot (12 kg soil capacity), for each of the seven treatment groups (9 pots/treatment group). Control seedlings were also grown in 9 pots with water application. IC50 NaCl for Jaya was calculated as 50 mM NaCl salt concentration under hydroponics (Supplementary Figure S5). The NaCl dose (9.5 g NaCl/per pot; equivalent to ~50 mM) was calculated considering the total water holding capacity of 12 kg paddy soil to be 3.25 L, which included soil saturation with water (1.85 L) and 5 cm standing water (1.4 L) required for inundation. Group-I(S) plants were treated with 9.5 g NaCl per pot in 1.4 L water at 7 days post-transplantation. Group-2 (T), -3 (F) and -4 (SS) seedlings were given NaCl treatment of similar magnitude at 22-, 50- and 75-days post-transplantation. For group 1–4, NaCl treatment was continued for 10 days with intermittent water application to maintain 5 cm standing water. Group-5 (S+T) seedlings were given NaCl treatment at 7 and 22 days, group-6 (S+T+F) seedlings at 7, 22 and 50 days and group-7 (S+T+F+SS) seedlings at 7-, 22-, 50- and 75-days post transplantation. For group 5, 6 and 7, NaCl treatment was continued for 10 days from the last application of saline solution. The crops were harvested at 10 days post the salt treatment. Whole plants (with root attached) were carefully uprooted from the soil (by flooding with water) so that the root system of the plants remained unaffected. For phenotyping analysis, five plants were randomly sampled per replication, with total 15 plants per treatment. For physiological and biochemical analysis, 3 plants were randomly sampled per replication with a total of 9 plants per treatment. For ionomic (cations and anions) analysis, the second fully expanded leaf of the main tiller and root samples were collected and dried. For analysis of different osmolytes, antioxidant enzymes and substrates, leaf and root samples were collected and snap chilled in liquid N_2_ and stored at −80 °C till further analysis.

### 2.2. Growth Parameters

After ten days of NaCl treatment at individual stage (group-1, -2, -3 and -4) or at the end of each combined-stage salinity stress (group-5, -6 and -7), 5 plants were selected per replication randomly (total 15 plants/group) and data pertaining to plant height, tiller number and leaf chlorophyll, was recorded. Leaf chlorophyll content was measured using SPAD Chlorophyll Meter (SPAD-502 plus-Konica Minolta, Konica Minolta Business Solutions Europe GmbH, Langenhagen, Germany). Differential phenotype was recorded for the individual plants in the treatments, along with their respective controls, for fresh weight of root and shoot. Dry weights of roots and shoots, were measured after drying the samples to a constant weight in an oven.

### 2.3. Determination of Macro- and Micro-Cations

For estimation of macro-cations (Na^+^, K^+^, Ca^+2^ and Mg^+2^) and micro-cations (Fe^+3^, Mn^+2^, Zn^+2^ and Co^+3^), leaf and root samples were dried in an oven at about 65 °C for 48 h, and then samples were ground in a grinding machine to pass through a 20-mesh sieve. Ground sample (0.5 g) was added to 10 mL di-acid mixture (HNO_3_:HClO_4_: 5:1) in a 100 mL digestion vessel. After an overnight incubation, samples were completely digested at 180 °C and finally volume was made up to 50 mL with double distilled water. A Continuum Source Flame Atomic Absorption Spectrometer (CSAAS 300, Analytik Jena, Jena, Germany) was used for the determination of Na^+^, K^+^, Ca^+2^, Mg^+2^ and Fe^+3^ [18]. Since the resonant wavelength differs for every element, the elements were quantified sequentially, one after the other. A calibration curve was made by plotting absorbance versus certified reference material (CRM) concentration. The concentration of the analyte in the sample was then calculated using the calibration curve as follows:

Concentration (in ppm or mg L^−1^) = (Absorbance of sample − y intercept)/slope of the calibration plot

Analysis of Mn^+2^, Co^+3^ and Zn^+2^ was done using VG PQ ExCell (Thermo Elemental, London, UK) Inductively Coupled Plasma Mass Spectrometer (ICP-MS) [19]. The instrument was calibrated with a series of certified reference materials (aqueous form) procured from E. Merck, Darmstadt, Germany, traceable to NIST. A plot of mass to charge ratio (e/m) versus integrated counts per second (ICPS) gave the calibration curve for the reference element, which was used for quantification of the analyte in the sample.

Concentration (in ppb or µg L^−1^) = (ICPS sample − y intercept)/slope of the calibration plot

### 2.4. Determination of Anions

For extraction of Cl^−^, SO_4_^2−^, and PO_4_^3−^, leaf and root samples were dried in an oven at about 65 °C for 48 h, and then samples were ground in a grinding machine to pass through a 20-mesh sieve. 0.10 g of oven-dried plant material was homogenized in porcelain mortar and pestle with deionized distilled water and then kept in water bath at 80 °C for 15 min. The samples were further sonicated for 1 h in a sonicator (Cole Parmar, Vernon Hills, IL, USA). The samples were centrifuged at 6000× *g* for 10 min and supernatants were filtered using Whatman No. 42 filter paper under suction. Ion exchange chromatography with conductometric detection in suppressed mode was used for the separation of different anions and their quantitative determination [20].

### 2.5. Estimation of Osmolytes Accumulation

Proline estimation was done in leaf and root tissues [21]. In short, 100 mg of leaf/root sample ground in liquid Nitrogen was mixed with 1 mL of aqueous sulfosalicylic acid (3%, *w*/*v*) and centrifuged at 10,000× *g* for 10 min. This reaction was set up by mixing 1 mL of this supernatant with equal volume of glacial acetic acid and ninhydrin reagent and incubated in boiling water bath for 1 h. Termination of reaction was done by snap chilling. After the reaction mixtures warmed to room temperature, phase separation was carried out through vigorous mixing with 2 mL toluene. The chromatophore-containing toluene was aspirated from the aqueous phase and absorbance was measured at 520 nm using toluene as blank. A standard curve of proline was generated and proline content was expressed as mg g^−1^FW.

For total soluble sugars (TSS) estimation, 100 mg of leaf/root tissue was homogenized with 10 mL of 80% ethanol. Sample were centrifuged at 10,000× *g* for 10 min and the reaction was set up by mixing 1 mL of the supernatant with 3 mL anthrone reagent (150 mg anthrone, 100 mL of 72% *w*/*w*). The reaction mixes were incubated at 100 °C in boiling water bath for 15 min, followed by termination of the reaction on ice. Absorbance was measured at 625 nm and soluble sugar contents were determined using glucose as standard. TSS content was expressed as mg g^−1^ FW [22].

### 2.6. Quantification of Malondialdehyde Content

For estimation of oxidative damage and lipid peroxidation, malondialdehyde (MDA) equivalents were quantified [23]. Briefly, 100 mg of leaf/root tissue was homogenized in liquid N_2_ and extracted in 1 mL of 0.1% trichloroacetic acid (TCA) solution. Following centrifugation at 12,000× *g* for 10 min, 0.5 mL of the supernatant was allowed to react with equal volume of thio-barbituric acid (TBA) solution. The reaction mixes were incubated in boiling water bath for 30 min and the reaction was terminated through snap chilling. Once the reaction mixture was warmed to room temperature, supernatant was collected by centrifugation at 12,000× *g* for 10 min at 25 °C. Absorbance of the clear supernatant was recorded at 532 nm and 600 nm and final concentration of MDA was expressed in μmol L^−1^ of MDA equivalents g^−1^FW.

### 2.7. Estimation of Enzymatic Antioxidants

To assay the activities of antioxidant enzymes, total protein was extracted by homogenization of leaf/root tissue in chilled extraction buffer containing 100 mM potassium phosphate buffer (pH 7.0), 0.1 mM ethylene diamine tetra acetic acid (EDTA) and 1% polyvinyl pyrrolidone (*w*/*v*) and quantified. The activity of superoxide dismutase (SOD), ascorbate peroxidase (APX) Guaiacol peroxidase (GPX) and catalase (CAT) were estimated from the same extract according to protocol by [24].

Total SOD (EC 1.15.1.1) activity was measured by the nitro blue tetrazolium (NBT) method at 560 nm. The enzymatic activity was calculated by adding 500 µL of 40 mM potassium phosphate buffer (pH 7.8), 330 µL distilled water, 50 µL of 13 mM Methionine, 75 µM NBT and 2 µM Riboflavin with 20 µL enzyme extract. SOD activity was expressed in unit mg^−1^ protein. One unit of SOD was defined as the amount of sample required to inhibit the rate of reduction of NBT by 50%.

For analysis of APX (EC 1.11.1.11), the extraction buffer also contained 2 mM ascorbate. The suspension was centrifuged (1700× *g*, 30 min, 4 °C) and the supernatant was used for assay of enzymatic activity in 20 µL enzyme extract. Total APX activity was measured by monitoring the decline in absorbance at 290 nm, as ascorbate (ε = 2.8 mM^−1^ cm^−1^) was oxidized for 3 min. APX activity was expressed in unit mg^−1^ of proteins.

For analysis of GPX (EC 1.11.1.7) activity, the reaction mixture contained 25 mM phosphate buffer (pH 7.0), 0.05% guaiacol, 10 mM H_2_O_2_ and 10 µL enzyme extract. Activity was determined by the increase of absorbance at 470 nm due to guaiacol oxidation (ε = 26.6 mM^−1^cm^−1^). GPX activity was also expressed in unit mg^−1^ of protein.

The CAT (EC 1.11.1.6) activity was measured using assay mixture of 3 mL consisted of 50 µL enzyme extract, 1.5 mL phosphate buffer (100 mM buffer, pH 7.0), 0.5 mL H_2_O_2_, and 0.95 mL distilled water. A decrease in the absorbance was recorded at 240 nm due to the decomposition of H_2_O_2_ (ε = 39.4 mM^−1^cm^−1^). The CAT activity was expressed as unit mg^−1^ of proteins.

### 2.8. Estimation of Non-Enzymatic Antioxidants

For estimation of oxidized (DHA; dehydroascorbate) and reduced ascorbate (ASC) contents, liquid N_2_ ground root/shoot samples (50 mg) were homogenized in 1 mL 6% trichloroacetic acid (TCA) under chilled conditions and centrifuged at 13,000× *g* for 5 min at 4 °C. To 200 μL of supernatant, 100 μL 75 mM phosphate buffer (pH 7.0) was added. In total ASC, 100 μL DTT (dithiothreitol; 10 mM) was added and incubated for 10 min at room temperature to reduce the pool of oxidized ASC. Then, 100 μL NEM (*N*-ethylmaleimide; 0.5%) was added to remove excess DTT. Along with this, 500 μL 10% TCA, 400 μL 43% orthophosphoric acid, 400 μL 4% 2,2′-bipyridyl, and 200 μL 3% FeCl_3_ were added to all the tubes. After incubation at 37 °C for 1 h, absorbance was measured at 525 nm. The level of DHA was calculated by subtracting ASC values from total ASC. The level of reduced glutathione (GSH) was determined fluorometrically using *o*–phthaldialdehyde (OPT) as a fluorophore. In brief, 0.10 g liquid N_2_ ground root/shoot sample was mixed with 1 mL of 0.1 M phosphate with 1 mM EDTA buffer (pH 8.0) and 25% meta phosphoric acid and spin at 20,000× *g* for 20 min at 4 °C. Supernatant was diluted 20-folds with phosphate buffer (pH 8.0). To final assay mixture, 0.05 mL of 0.1% OPT was added. After thorough mixing and incubation at room temperature for 15–20 min, the fluorescence intensity was monitored at 420 nm after excitation at 350 nm (20 °C) [25].

### 2.9. Statistical Analysis

The experiments were conducted in randomized block design (RBD) with three experimental replicates. For phenotypic evaluation, data were collected from randomly selected 5 plants/replication (15 plants/treatment). For studying the ionomic and biochemical parameters, data were collected from 3 randomly selected plants/replication. Since all the data readings were of different scales, fold change was calculated for statistical analysis. All fold change data was converted to Log2 scale to have a better representation. One-way analysis of variance (ANOVA) was performed with the whole dataset to confirm the variability of data and validity of results, and Duncan’s multiple range test (DMRT) was performed to determine the significant difference between treatments. Different letters in the graphs indicate significantly different values (DMRT, *p*  ≤  0.05). Principal component analysis (PCA) was performed with whole datasets which included 7 treatment conditions, using Origin 2016 (Origin Lab, Northampton, MA, USA), and the first two components (PC1 and PC2) explaining the maximum variance in the datasets were used to make biplots.

## 3. Results

### 3.1. Phenotypic Evaluation of CSR36 and Jaya under Stage-Specific Salinity

**Figure 2:**
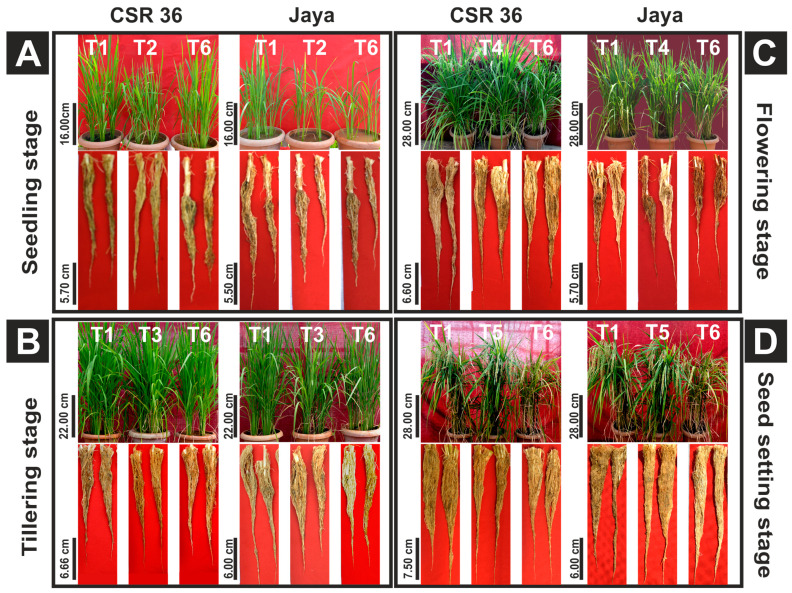
Shoot and root phenotypes of CSR36 and Jaya under stage-specific and combined-stage salinity stress 10 days post salt treatment. Rice seedlings were grown in polyhouse under control condition and subjected to 50 mM NaCl salinity stress at stage-specific; seedling (S), tillering (T), flowering (F), seed setting (SS), and combined-stages; seedling + tillering (S+T), seedling + tillering + flowering (S+T+F), seedling + tillering + flowering + seed setting (S+T+F+SS), for 10 days. Qualitative shoot and root images were taken at (**A**) seedling stage, (**B**) tillering stage, (**C**) flowering stage and (**D**) seed setting stage 10 days post salt treatment. (**Notations; Seedling stage:** T1 Control, T2 S-stage salinity, T6 Combined stage salinity (S+0-stage); **Tillering stage:** T1 Control, T3 T-stage salinity, T6 Combined stage Salinity (S+T-stage); **Flowering stage:** T1 Control, T4 F-stage salinity, T6 Combined stage Salinity (S+T+F-stage); **Seed setting stage:** T1 Control, T5 SS-stage salinity, T6 Combined stage Salinity (S+T+F+SS-stage). Side bar indicates scale for CSR36 and Jaya in shoot and roots.).

**Figure 3:**
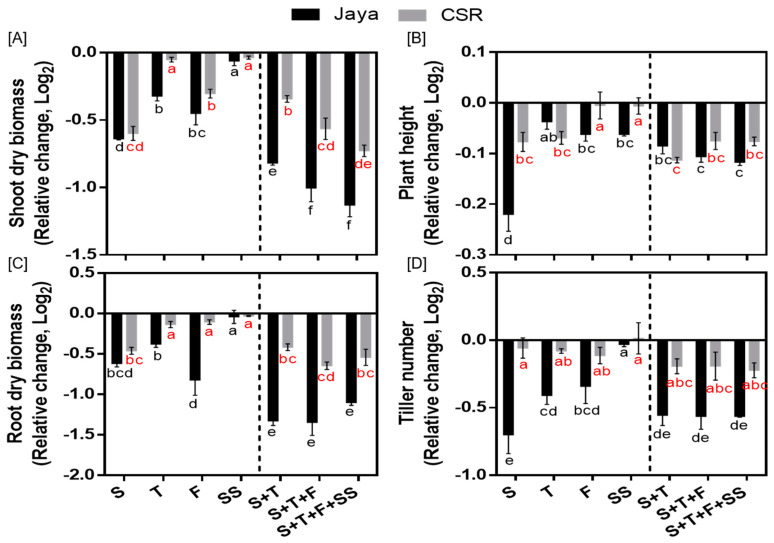
10 Days post treatment phenotyping of rice varieties Jaya and CSR36 under stage−specific and combined−stage salinity stress. Rice seedlings were grown in polyhouse under control condition and subjected to 50 mM NaCl salinity stress at stage−specific; seedling (S), tillering (T), flowering (F), seed setting (SS), and combined−stages; seedling + tillering (S+T), seedling + tillering + flowering (S+T+F), seedling + tillering + flowering + seed setting (S+T+F+SS), for 10 days. Differential phenotyping was quantified in terms of shoot dry biomass (**A**), root dry biomass (**B**), plant height (**C**) and tiller number (**D**). Refer to Supplementary Table S1 for statistics of different plant phenotype data at different time points. All the values are mean of triplicates ± SD. Different letters in black and red colors indicate significantly different values across treatments for Jaya and CSR 36 respectively (DMRT, *p* ≤ 0.05), considering the fold change in both Jaya and CSR36 together. A dashed line separates stage specific salinity (to the left) and combined stage salinity (to the right) treatments.

The differential phenotyping of rice seedlings revealed significant differences in CSR36 and Jaya under stage-specific as well as combined-stages salinity stress (Figure 2A–D). Jaya exhibited significant reduction at S-stage salinity stress as indicated by reduction to 0.50-, 0.64-, 0.85- and 0.61-fold in shoot fresh and dry biomass, plant height and tiller number, respectively, compared with those of control (Figure 3A–D; Supplementary Table S1). Among adult plant stages (T-, F- or SS-) in Jaya, F-stage showed comparatively higher sensitivity to saline conditions as shoot and root dry biomass and chlorophyll content decreased to 0.73-, 0.57- and 0.90-fold as compared with those of control (Figure 3A,B; Supplementary Table S1). Although, salt-sensitivity was comparable in CSR36 and Jaya at S- and SS-stages (Figure 3A,C), CSR36 showed relatively higher salt-tolerance phenotype at both F- and T-stages, as indicated by 1.63- and 1.12-fold increase in root dry biomass and chlorophyll content, respectively compared with those of Jaya (Figure 3C; Supplementary Table S1). In combined-stage salinity stress, extent of damage was similar in all three treatment groups viz., S+T-, S+T+F- and S+T+F+SS-stages and were non-significant both in CSR36 and Jaya. However, CSR36 exhibited lower sensitivity in all three treatments as compared with Jaya, indicating higher tolerance of CSR36 to multi-stage salinity stress (Figure 3A–D).

### 3.2. Enhanced Antioxidative Defense Helps CSR36 Tolerate Salinity Stress

**Figure 4:**
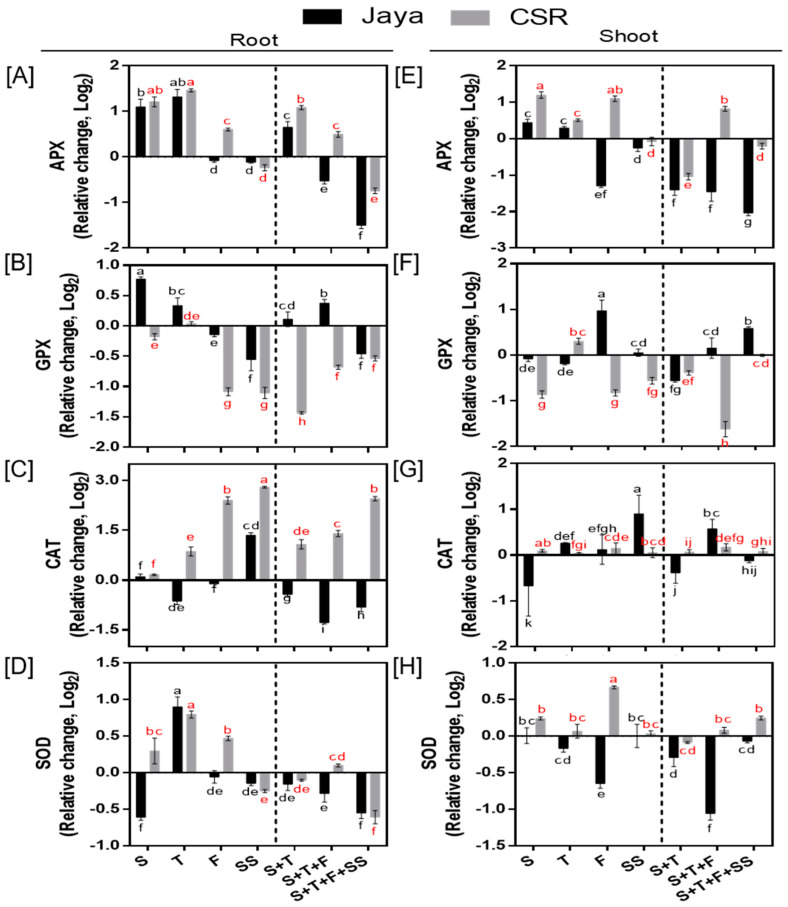
Spatial and temporal response of antioxidant enzymes in Jaya and CSR36. The rice seedlings were subjected to 50 mM NaCl salinity stress for 10 days at S− (seedling), T− (tillering), F− (flowering), SS− (seed setting) under stage-specific and S+T− (seedling + tillering), S+T+F− (seedling + tillering + flowering), S+T+F+SS− (seedling + tillering + flowering + seed setting), under combined-stage salinity stress. After 10 days of treatment, the root and shoot tissues were harvested and analyzed for ascorbate peroxidase (APX; (**A**,**E**)), glutathione peroxidase (GPX; (**B**,**F**)), catalase (CAT; (**C**,**G**)), and superoxide dismutase (SOD; (**D**,**H**)). The data are represented in the form of relative change with respect to respective control and are converted to Log2. Refer to Supplementary Table S2 for statistics of different antioxidant enzyme data at different time points. Different letters in black and red colors indicate significantly different values across treatments for Jaya and CSR 36 respectively (DMRT, *p* ≤ 0.05), considering the fold change in both Jaya and CSR36 together. A dashed line separates stage specific salinity (to the left) and combined stage salinity (to the right) treatments.

CSR36 exhibited increased APX and CAT activity under salt stress in CSR36. At F-stage salinity, APX activity significantly increased by 1.51- and 2.13-fold in root and shoot, respectively, as compared with those of Jaya (Figure 4A,E). Similar increased activity in APX was also seen under combined-stage salinity in CSR36. Among three tested combined-stage treatments, APX activity in roots of CSR36 was increased by 2.12- and 1.41-fold under S+T- and S+T+F-stages, respectively compared to those of control (Figure 4A), which coincided with reduced GPX activity (Figure 4B,F). On the contrary, APX and GPX activities were significantly lower and higher, respectively in Jaya at all tested combined-stage treatments (Figure 4A,B,E,F). In general, the activities of CAT (in root) and SOD (in both root and shoot) were increased in CSR36 under stage-specific salinity. The maximal increase in CAT activity was seen by 5.67-, 2.71-fold at F- and SS-stage and 6.45- and 9.58-fold at S+T+F- and S+T+F+SS-stages, respectively in CSR36 roots, compared with those of Jaya (Figure 4C). At S- and F-stages, SOD activity was increased by 1.83- and 1.43-fold in root respectively, (Figure 4D) and 2.52-fold at F-stage in shoot (Figure 4H) compared with those of Jaya.

### 3.3. Modulation of Cellular Redox State in CSR36

**Figure 5:**
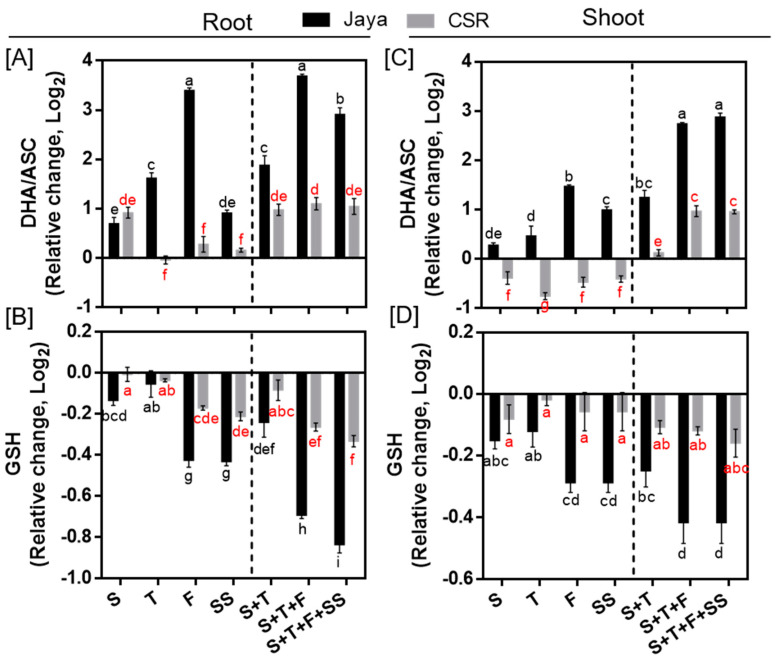
Spatial and temporal response of antioxidant substrates in Jaya and CSR36. The rice seedlings were subjected to 50 mM NaCl salinity stress for 10 days at S− (seedling), T− (tillering), F− (flowering), SS− (seed setting) under stage-specific and S+T− (seedling + tillering), S+T+F− (seedling + tillering + flowering), S+T+F+SS− (seedling + tillering + flowering + seed setting), under combined-stage salinity stress. After 10 days of treatment, the root and shoot tissues were harvested and analyzed for ascorbate oxidized/reduced (DHA/ASC; **A**,**C**), and reduced glutathione (GSH; **B**,**D**). The data are represented in the form of relative change with respect to respective control and are converted to Log2. Refer to Supplementary Table S2 for statistics of different antioxidant substrate data at different time points. Different letters in black and red colors indicate significantly different values across treatments for Jaya and CSR 36 respectively (DMRT, *p* ≤ 0.05), considering the fold change in both Jaya and CSR36 together. A dashed line separates stage specific salinity (to the left) and combined stage salinity (to the right) treatments.

Under NaCl stress, a significant increase in the level of DHA/ASC ratio in both root and shoot tissues of Jaya was observed at all salinity treatment stages indicating oxidative damage (Figure 5A,C). Maximum increase in DHA/ASC ratio was observed in Jaya roots at F- and S+T+F-stages by 10.53- and 12.95-fold, respectively compared with those of controls (Figure 5A). Unlike Jaya, CSR36 maintained a lower DHA/ASC ratio under different salinity treatments in both the tissues (Figure 5A,C). Although, GSH level declined at most of the tested conditions in Jaya, the decrease was more significant at F- and SS- stages to 0.74-fold each in roots, which further decreased at S+T+F and S+T+F+SS salinity to 0.61- and 0.56-fold, in root (Figure 5B) and 0.75-fold each, respectively, in shoot (Figure 5D), compared with those of respective controls. The extent of GSH reduction was relatively lower in CSR36 roots, as indicated by reduction to 0.85-fold each at F- and SS-stage and 0.79- and 0.83-fold reduction at S+T+F and S+T+F+SS in roots compared with those of respective controls (Figure 5B). No significant reduction was noticed in GSH level in CSR36 shoot, across any of the tested salinity treatments (Figure 5D).

### 3.4. Effect of Salt Induced Toxicity on Osmotic Adjustment and Lipid Peroxidation

**Figure 6:**
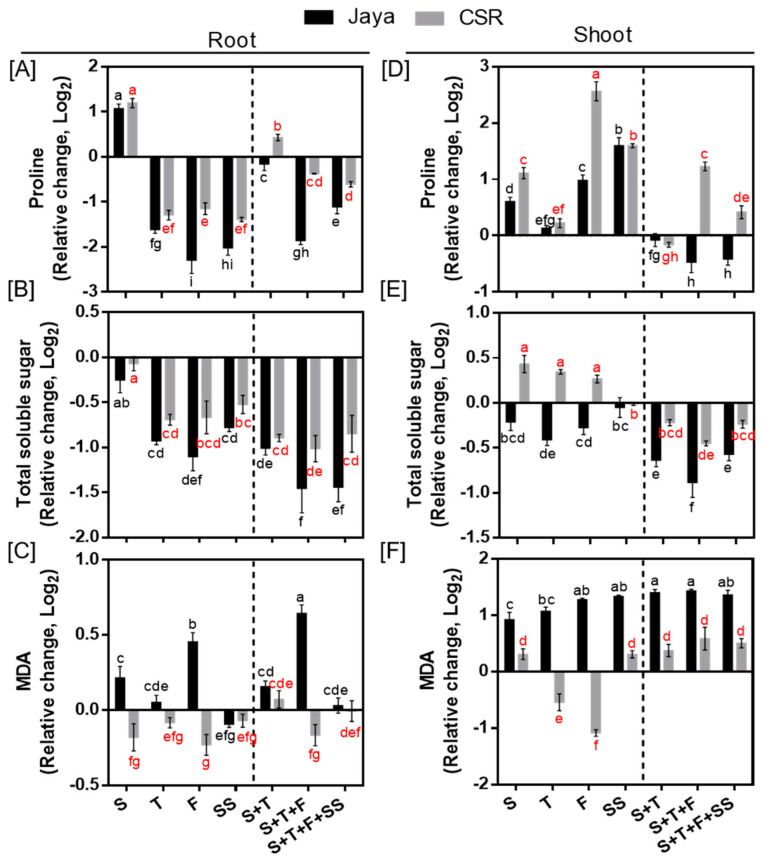
Spatial and temporal response of osmolytes and lipid peroxidation in Jaya and CSR36. The rice seedlings were subjected to 50 mM NaCl salinity stress for 10 days at S− (seedling), T− (tillering), F− (flowering), SS− (seed setting) under stage-specific and S+T− (seedling + tillering), S+T+F− (seedling + tillering + flowering), S+T+F+SS− (seedling + tillering + flowering + seed setting), under combined-stage salinity stress. After 10 days of treatment, the root and shoot tissues were harvested and analyzed for Proline (**A**,**D**), total soluble sugar (TSS; **B**,**E**) and malonaldehyde (MDA; **C**,**F**). The data are represented in the form of relative change with respect to respective control and are converted to Log2. Refer to Supplementary Table S3 for statistics of different osmolytes and MDA data at different time points. Different letters in black and red colors indicate significantly different values across treatments for Jaya and CSR 36 respectively (DMRT, *p* ≤ 0.05), considering the fold change in both Jaya and CSR36 together. A dashed line separates stage specific salinity (to the left) and combined stage salinity (to the right) treatments.

Proline and TSS accumulation decreased in roots of Jaya and CSR36 at all the tested salinity treatments; however, the decrease was more significant in Jaya (Figure 6A,B). However, at S- and S+T-stage, 1.1- and 0.4-fold increase in proline accumulation was registered in CSR36 root. In contrast, enhanced accumulation of proline and TSS was seen in CSR36 shoots, particularly, of proline, which increased by 2.30- and 1.58-fold at F-and SS-stage and 1.48-, 2.85- and 1.41-fold at S+T, S+T+F and S+T+F+SS-stages, respectively, compared with those of Jaya (Figure 6A). Like roots, TSS levels were affected significantly in Jaya shoots with reduced accumulation at all treatment stages indicating poor osmotic adjustment in shoots as well (Figure 6D). MDA levels were increased by 1.37- and 2.70-fold at F- and S+T+F-stage in roots and a minimum 1.80-fold increase at all treatment stages in shoots of Jaya, compared with their respective controls (Figure 6C,F). No significant change in MDA levels was seen in CSR36, in any of the tested salinity treatments (Figure 6C,F).

### 3.5. Effect of Salt Stress on Macro- and Micro-Cations

Under all the tested stages, the Na^+^ accumulation in Jaya was significantly higher than CSR36. At F-, SS- and S+T+F+SS stages, Jaya accumulated 2.25-, 2.03-, 3.50-fold Na, respectively, in root; while, in case of CSR36, these changes were limited to only 0.54-, 0.49-, 0.86-fold, respectively, compared to their respective controls (Table 1, Figure S1A). A similar trend was also seen in case of shoot (Figure S1B), which was in accordance with relatively tolerant phenotypes of CSR36 than Jaya, at these salinity treatment stages (Figure 2 and Figure 3). Root K^+^ and Ca^+2^ accumulation exhibited a positive bias; however, no significant differences were observed between the roots of CSR36 and Jaya, at any of the tested salinity treatments (Figure S1A). In contrast, the shoot K^+^ and Ca^+2^ showed higher accumulation in CSR36 at all stages, in particular at F-stage with 1.19- and 1.40-fold increase compared with those of Jaya (Table 1, Figure S1B). Mg^+2^ levels followed a similar trend in both the varieties.

Preferential Na^+^ uptake and accumulation in Jaya root led to marked increase in Na^+^/K^+^, Na^+^/Ca^+2^ and Na^+^/Mg^+2^ ratios across all treatment conditions with highest increase by 4.58-, 4.66- and 4.14-fold, respectively, compared with those of CSR36 at F-stage (Table 1, Figure S2A). CSR36 roots exhibited significantly lower cationic ratios at all treatment stages, except S-stage, at which higher Na^+^/K^+^ (1.77-fold), Na^+^/Ca^+2^ (1.88-fold) and Na^+^/Mg^+2^ (1.43-fold) ratios were observed, compared with those of control (Table 1). Like root tissues, Jaya exhibited preferential transport of Na^+^ over K^+^ and Ca^+2^ leading to significantly higher Na^+^/K^+^, Na^+^/Ca^+2^ and Na^+^/Mg^+2^ ratios in shoot at all treatment stages (Table 1). Although, CSR36 had comparable ratios like control across all the stages; however, significant reduction in leaf Na^+^/K^+^ and Na^+^/Ca^+2^ ratio was observed at S+T-stage by 3.62- and 2.79-fold and at S+T+F+SS-stage by 1.98- and 1.81-fold, respectively, compared with those of Jaya (Table 1, Figure S2B).

The accumulation of Fe^+3^, Mn^+2^, Zn^+2^ and Co^+3^ in roots and shoots was negatively impacted across different stage-specific salinity treatments in Jaya making it susceptible to NaCl induced salt toxicity (Table 2, Figure S3A,B). Except for S-stage salinity, Fe^+3^, Mn^+2^ and Co^+3^ accumulation in roots and Mn^+2^ accumulation in leaf of CSR36 was significantly higher at all other treatment stages as compared with Jaya (Table 2). Zn^+2^ accumulation was poor in both the varieties under different salinity treatments, however, at F-stage Zn^+2^ accumulation showed significant increase by 1.78- (roots) and 1.44- (shoots) fold in CSR36 compared with those of Jaya (Table 2, Figure S3A,B).

### 3.6. Effect of Salt Stress on Anions

The accumulation of Cl^−^ and SO_4_^2−^ was significantly lower in CSR36 roots and shoots at most of the treatment stages as compared with that of Jaya (Figure S4A,B). In general, Cl^−^ toxicity was more severe in roots than in shoots and combined-stage salinity treatments were found to be more sensitive particularly in Jaya. In addition, shoot accumulation of SO_4_^2−^ in Jaya was significantly higher which further led to increase the impact of NaCl induced toxicity. CSR36 exhibited reduced Cl^−^ accumulation at F- and T-stage by 2.66- and 2.50-fold, respectively in roots alongside, 1.28-fold lower Cl^−^ accumulation at F-stage in leaves, compared with those of Jaya (Figure S4A,B). At S-stage, 1.99- and 0.78-fold higher Cl^−^ accumulation was observed in CSR36 and Jaya root, respectively (Figure S4A). No significant increase in shoot Cl^−^ content was seen in CSR36 leaves at stage specific salinity (Figure S4B). At combined-stage salinity stress specially at S+T+F+SS and S+T, Cl^−^ accumulation was reduced by 4.80- and 2.06-fold in CSR36 roots, respectively, compared with those of Jaya (Figure S4A) which further dissipated in leaves at respective stages (Figure S4B). PO_4_^3−^ accumulation did not exhibit any clear trend; however, it was found to be reduced across all the tested treatment conditions (Figure S4A,B).

### 3.7. Understanding Treatment-Variable Interactions through PCA-Based Clustering

**Figure 7:**
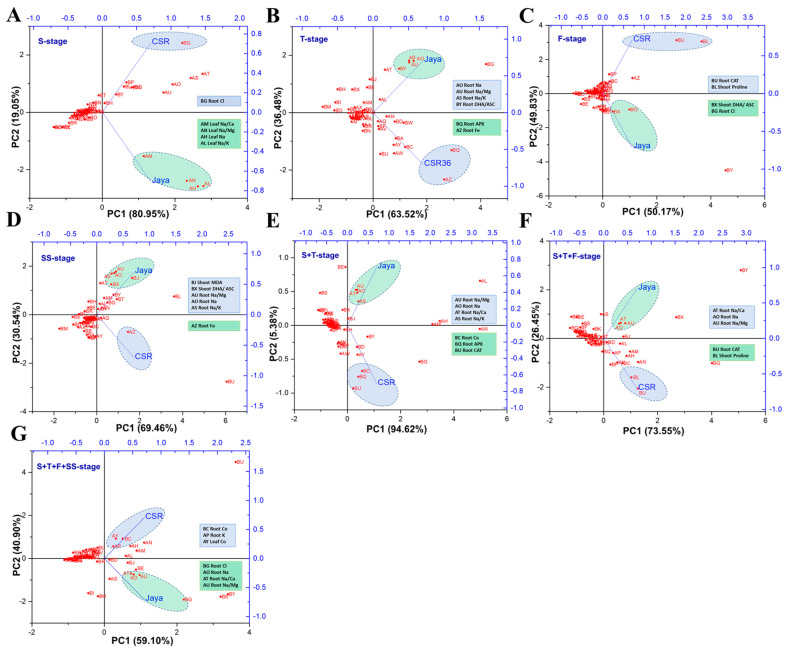
Principal component analysis on field grown plants to understand treatment-variable interaction. The principal component analysis (PCA) was performed to identify the variables associated with different treatments including Jaya (50 mM NaCl per pot) and CSR36 (50 mM NaCl per pot). The biplots were generated independently for stage−specific salinity i.e., S−stage (**A**), T−stage (**B**), F−stage (**C**), SS−stage (**D**) and combined−stage salinity i.e., S+T−stage (**E**), S+T+F−stage (**F**), S+T+F+SS−stage (**G**). Individual PCA plots helps in identifying key variables responsible for salinity phenotype in Jaya and CSR36. (Refer to Supplementary Figure S6 for key variables regulating salinity response in rice varieties Jaya and CSR36 at stage-specific and combined-stage salinity).

Principal Component Analysis (PCA) allowed easy visualization of complex data by treatment-variable association in a stage-specific manner. Contrasting phenotypes were observed between Jaya and CSR36 at different stages which were best described by PC1 with contributions ranging from 94.62% (S+T-stage) to 50.17% (F-stage). Higher root Na^+^ and associated cationic ratios such as Na^+^/Ca^+2^, Na^+^/Mg^+2^ and Na^+^/K^+^ were identified as major contributors associated with salt-sensitive nature of Jaya, at most of the treatment stages (Figure 7B–G), except at S-stage, wherein Na^+^ accumulation in shoots was identified as a major driver for sensitive phenotype (Figure 7A). The reduced salt-toxicity in CSR36 was attributable to efficient management of the redox status by different antioxidant enzymes (T-, F-, S+T-, S+T+F-stages; Figure 7B,C,E,F), maintenance of osmotic balance prominently by proline accumulation (F- and S+T+F-stages; Figure 7C,F) and micro-cation balance with high root Fe (T- and SS-stage; Figure 7B,D) and high root Co (S+T- and S+T+F+SS-stages; Figure 7E,G).

## 4. Discussion

### 4.1. NaCl-Induced Toxicity Is Developmental Stage-Specific

Salinity tolerance at seedling stage does not impart tolerance at reproductive stages in rice [26]; hence, proper phenotypic analysis at different stages is necessary to achieve higher yield under salt stress. Sensitivity to salt stress was higher at seedling stage as indicated by significant reduction in shoot and root dry biomass (Figure 2 and Figure 3A,B) in both the tested varieties. The poorly developed root and shoot (2–3 leaf stage) system at seedling stage might cause higher degree of osmotic and ionic stress, resulting in water deficit and reduced shoot and root biomass [27]. However, adult plant tolerance is different from seedling stage and is regulated by an independent set of genes [28]. Based on stage-specific phenotyping data, salt sensitivity in Jaya at different stages was ranked as S > F > T > SS. In contrast, CSR36 exhibited tolerance at all three adult plant stages sequentially as SS ≥ F > T stage. Combined-stages salinity stress did not exhibit any significant differences in the toxicity induced in CSR36, which confirms the importance of early vegetative and tillering stages in determining the plants’ overall phenotype, till maturity, under salt stress (Figure 3A–D). Hence, CSR36 can be used as a donor for tolerance at adult plant and combined-stage salinity stress. Root indices appeared to be a better selection parameter for salt tolerance which operates through maintenance of better shoot growth possibly through dilution of salt or salt exclusion by roots, limiting Na^+ ^ion accumulation in the shoots resulting more vigorous shoot growth [29].

### 4.2. Antioxidant Defense and Osmotic Adjustment Reduced NaCl-Induced Toxicity

Salinity stress induces excessive production of ROS including H_2_O_2_ and O_2_^.-^ owing to ionic imbalance and oxidative stresses. Although plants possess an efficient antioxidative defense; however, the lack of coordination results in increased ROS levels and oxidative damage [30]. SOD constitutes the first line of enzymatic defense under salt induced oxidative stress [31], as superoxide molecule is produced primarily under any kind of stress which converts superoxide molecules to hydrogen peroxides. Further, APX and CAT enzymes are involved in catabolizing hydrogen peroxides into H_2_O and O_2_ in plants. Higher activities of SOD (shoot), CAT (root) and APX (root and shoot both) strengthen antioxidant potential at combined-stage salinity in CSR36 compared with that of Jaya, which coincides with earlier observation wherein increased SOD, APX and CAT activities have been shown to contribute to enhanced salt tolerance [32,33]. PCA analysis validated CAT and APX activities in root as the major drivers for salt tolerance in CSR36 (Figure 7C,E,F). GPX activity was relatively lower in tolerant cultivar CSR36 as opposed to CAT and APX. GPX is reported to be localized in various subcellular compartments like cell wall, cytosol, vacuole and extracellular spaces and actively participates in various developmental processes, wound healing, cell wall biosynthesis, ethylene signalling etc. GPX was also reported as an intrinsic defense mechanism to counter oxidative damage in rice plants and correlated with increased release of peroxidases localized in the cell walls [34]. Decreased activity of the GPX in CSR36 may be due to a tradeoff with inherent potential of antioxidant defense pool associated with active involvement of CAT and APX to check the peroxides leading to lower lipid peroxidation resulting low MDA levels and better redox management under salt stress. The results are in coherence with earlier reports where GPX activity was found to be unchanged/decreased in tolerant cultivars under salt stress [35]. In addition, the non-enzymatic antioxidant substrates (ASC/DHA and GSH) play important roles in supporting antioxidant defense machinery and maintenance of redox balance in plants [36]. Interestingly, CSR36 maintained lower levels of DHA/ASC ratio and higher levels of GSH as compared with Jaya at most of the developmental stages in both root and shoots indicating better redox management (Figure 5A–D). This might have also resulted in lowering the levels of MDA (Figure 6C,F), indicating low oxidative damage. Similar observations were recorded at the reproductive stage in rice [37] and at seedling stage in maize [38]. Cellular osmotic management is of utmost importance to maintain water balance under different abiotic stresses including salt stress. Higher accumulation of proline and TSS at F-stage and combined-stage salinity was observed in CSR36, which contributes to the alleviation of salt-induced osmotic disturbances. Under hyper-osmotic conditions (e.g., salinity and drought stress), root growth and root-water confluence area are significantly limited thus limiting the water uptake and resulting in sensitivity under salinity stress [39]. Thus, higher root biomass at critical growth stages i.e., F- and T- stages in stage specific and all 3 combined stage salinity stress in CSR36 (Figure 3C) could be seen as the manifestation of simultaneous management of osmotic and oxidative stress.

### 4.3. Ionic Homeostasis Contributes to Salt Tolerance in CSR36

Ionic homeostasis is an important strategy, adapted by most of the plants to mitigate salt induced toxicity [9]. Among different complexities of stress tolerance mechanisms, maintaining an optimal cytosolic Na^+^/K^+^, Na^+^/Mg^+2^ and Na^+^/Ca^+2^ ratio is considered to be the most critical [40]. Although both the tested genotypes exhibited sensitivity at early seedling stage owing to high Na^+^ accumulation and thereby resulting toxic levels of Na^+^/K^+^, Na^+^/Mg^+2^ and Na^+^/Ca^+2^ in roots and shoot tissues, CSR36 still maintained relatively higher biomass and tiller number as compared with Jaya (Table 1, Figure S2A,B). In the adult plant stage, CSR36 either maintained a comparable (shoot) or lower level (root) of Na^+^ under different stages of salinity as compared with that of Jaya, resulting in better ionic homeostasis (Supplementary Figures S1 and S2A). The inherent Na-tolerance in CSR36 might be associated with efficient Na^+^ exclusion in roots, however, it demands future investigation. In addition to root, Na^+^ levels in Jaya shoot were either comparable under stage specific salinity or significantly higher under combined stage salinity as compared with that of CSR36 which might have resulted in reduced biomass and tiller number. In contrast, shoot accumulated relatively low K^+^ with higher accumulation Na^+^ in Jaya as compared with CSR36 which resulted in higher Na^+^/K^+^ ratio at all stages (Table 1, Figures S1 and S2B). Higher transport of K^+^ and Ca^+2^ over Na^+^ in CSR36 even with constant K^+^ and Ca^+2^ uptake in both the varieties suggested ionic-discrimination as a key strategy operating in CSR36 (Table 1, Supplementary Figure S1A,B). In addition to restricted Na^+^ uptake, CSR36 was also able to minimize the upward movement of Na^+^ from root to mesophyll tissues. This might be due to superior xylem unloading and/or vacuolar sequestration. Na^+^-induced chlorosis was also lower in CSR36 at later stages of salinity, indicating better tissue tolerance for Na^+^ that helps to maintain chlorophyll synthesis and ultimately chlorophyll concentration [41]. Thus, salt tolerance of CSR36 at adult plant stages and combined-stages was explained on the basis of efficient Na^+^-exclusion and better ionic discrimination in transporting K^+^ and Na^+^ from root to shoot.

### 4.4. NaCl-Induced Disequilibrium in Micro-Cations and Anions

The concentration and solubility of micronutrients usually changes under saline conditions but it may or may not affect their uptake by plants [42]. However, the effect of salinity on uptake of micronutrients and distribution in roots and shoots are poorly understood. In the present experiment, Fe^+3^ and Mn^+2^ content increased significantly in adult plant salinity and remained unaffected during combined-stages in root and shoot tissues of CSR36; however, the translocation of Fe^+3^ in the shoots was relatively low as compared with Mn^+2^ which suggests ion selectivity under salt stress in tolerant plants (Table 2, Figure 3A,B). High salinity caused by saline and alkaline stress reduces the solubility of Fe^+3^ in solution, leading to chlorosis which was visible in Jaya under salt stress (Figure S3B) [43]. Compared to Fe^+3^ and Mn^+2^, Zn^+2^ uptake was significantly reduced in Jaya root and shoot tissues at critical stages i.e., F-stage in stage specific and S+T+F- and S+T+F+SS-stage in combined-stage salinity stress but CSR36 exhibited significantly higher Zn^+2^ uptake and better translocation at these stages in shoots as compared with Jaya, indicating ion selectivity (Table 2, Figure S3A,B). Low accumulation of Zn^+2^ under salt stress could be attributed to decreased expression of Zn-transporters involved in zinc uptake [44], zinc translocation [45] and vacuolar sequestration [46], as shown previously. Application of Zn under salt stress improves seed germination, seedling growth, plant water relations, water uptake, and nutrient homeostasis, therefore improving plant growth. In addition, it also protects the photosynthetic apparatus from oxidative stress and improves chlorophyll synthesis, stomatal movement, carbon fixation, and hormone and osmolytes accumulation. Moreover, Zn application has been seen to increase the synthesis of secondary metabolites and stress responsive gene expression resulting higher antioxidant activities to counter the toxic effects of salt stress [47]. Among all the tested micro cations, Co^3+^ have been found to be a critical component imparting tolerance to salinity in CSR36 with enhanced uptake both in root and shoot particularly at F-stage and combined-stage salinity stress (S+T+F- and S+T+F+SS-stage) which was further supported by PCA (Table 2, Figure 7C and Figure S3A,B). Co^3+^ is known to regulate plant-water homeostasis [48], hence its higher accumulation in CSR36 could provide the survival benefit under salt stress conditions.

Like cations, anions (Cl^−^, SO_4_^2−^ and PO_4_^3−^) can also cause ionic imbalance of essential elements including N, P and S leading to enhanced damage to plants [14]. Cl^−^ act as a non-specific osmotic agent and hence is accumulated in higher concentrations over PO_4_^3−^ or SO_4_^2−^ under salt stress [49]. High Cl^−^ uptake and transport to shoots were observed in Jaya resulting in a sensitive phenotype both in stage-specific and combined-stage salinity stress. Barring S-, T- and S+T+F-stage, CSR36 exhibited significantly low Cl^−^ accumulation in roots and transport low Cl^−^ and high PO_4_^3−^ to the shoots at critical growth stages (F-, S+T- and S+T+F+SS-stages) as compared with Jaya indicating ion selectivity and discrimination (Figure S4A,B) [40]. The low shoot Cl^−^ concentration is maintained by restricting xylem-driven root to shoot transport (i) by higher Cl^−^ efflux from the root to soil solution or (ii) by reducing the xylem loading as evident in CSR36 [50]. However, SO_4_^2−^ and PO_4_^3−^ are probably assimilated in plant metabolism [51] resulting enhanced plant growth and reduced internal concentration in roots and shoots as seen in the case of tolerant variety CSR36 under salt stress.

Thus, CSR36 maintained lower levels of Na^+^ and Cl^−^, in particular at F-stage and combined-stage salinity, without lowering Fe^+3^, Mn^+2^ and Co^+3^ accumulation in root and transport to the shoots, indicating its ability to maintain ionic homeostasis between essential and non-essential ions responsible for higher plant biomass under salt stress.

## 5. Conclusions

Taken together, the present study highlighted that salinity tolerance manifests in growth stage-specific manner and tolerance at one stage is controlled independently of tolerance at other stages. Seedling stage was found to be the most sensitive stage for salinity followed by flowering stage; however, phenotypic data suggested no significant changes in case of combined-stage salinity stress. CSR36 exhibited higher tolerance at adult plant stage (SS ≥ F > T) and combined-stage salinity stress. This also confirmed the tolerance of CSR36 towards saline soil (earlier known to tolerate alkaline soil) dominated by NaCl salt. To the best of our knowledge, this is the first report on the effect of stage-specific salinity stress in rice, which is generally experienced in coastal areas. The greater tolerance of the CSR36 was also supported by higher antioxidative potential, improved osmotic adjustment and better homeostasis between essential vs. non-essential nutrients. Thus, a balance of redox and ionic homeostasis regulations helps to manage oxidative and ionic stress associated with stage specific and combined stage salinity in rice [52]. Understanding and managing these interrelated processes are crucial for developing strategies to enhance salt tolerance in plants. Additionally, the findings highlight the significance of stage-specific salinity response, which needs to be considered while designing future rice improvement programs for enhanced salt tolerance.

## Figures and Tables

**Table 1:** Effects of 50 mM NaCl stress in rice varieties Jaya and CSR36 on log_2_ fold change of macro-cations (Na^+^, K^+^, Ca^+2^ and Mg^+2^) uptake and their ratios (Na^+^/K^+^, Na^+^/Ca^+2^ and Na^+^/Mg^+2^) in root and leaf in stage-specific and combined-stage salinity stress.

Macro Cations				
	Root		Shoot	
Treatments	Jaya	CSR36	Jaya	CSR36
Na^+^ levels				
S	2.16 ± 0.131 ^a^	1.583 ± 0.084 ^c^	3.844 ± 0.099 ^a^	0.445 ± 0.188 ^def^
T	1.829 ± 0.124 ^b^	0.098 ± 0.027 ^f^	0.349 ± 0.129 ^fg^	0.087 ± 0.224 ^ghi^
F	1.174 ± 0.006 ^d^	−0.876 ± 0.016 ^h^	0.42 ± 0.075 ^efg^	0.062 ± 0.006 ^ghi^
SS	1.027 ± 0.015 ^d^	−1.026 ± 0.057 ^h^	0.251 ± 0.025 ^fgh^	−0.184 ± 0.023 ^i^
S+T	2.109 ± 0.049 ^a^	0.327 ± 0.063 ^e^	3.352 ± 0.182 ^b^	1.812 ± 0.231 ^c^
S+T+F	1.762 ± 0.02 ^bc^	0.274 ± 0.032 ^e^	0.794 ± 0.046 ^de^	0.903 ± 0.004 ^d^
S+T+F+SS	1.811 ± 0.013 ^b^	−0.218 ± 0.036 ^g^	0.516 ± 0.116 ^def^	−0.117 ± 0.112 ^hi^
K^+^ levels				
S	−0.208 ± 0.05 ^de^	−0.193 ± 0.028 ^cde^	−0.025 ± 0.066 ^de^	−0.089 ± 0.027 ^def^
T	0.045 ± 0.051 ^bcd^	0.093 ± 0.035 ^bc^	−0.551 ± 0.033 ^i^	0 ± 0.031 ^cde^
F	0.037 ± 0.039 ^bcd^	0.181 ± 0.024 ^b^	−0.12 ± 0.025 ^efg^	0.15 ± 0.015 ^bc^
SS	−0.431 ± 0.075 ^ef^	−0.573 ± 0.241 ^f^	−0.253 ± 0.027 ^gh^	0.041 ± 0.102 ^cd^
S+T	0.095 ± 0.054 ^bc^	−0.151 ± 0.106 ^cde^	−0.543 ± 0.037 ^i^	−0.218 ± 0.09 ^fgh^
S+T+F	0.257 ± 0.056 ^b^	0.689 ± 0.013 ^a^	−0.291 ± 0.023 ^h^	0.262 ± 0.012 ^b^
S+T+F+SS	0.217 ± 0.116 ^b^	−0.233 ± 0.107 ^de^	0.136 ± 0.056 ^bc^	0.441 ± 0.021 ^a^
Ca^+2^ levels				
S	−0.297 ± 0.121 ^d^	−0.298 ± 0.038 ^d^	0.768 ± 0.032 ^a^	0.3 ± 0.015 ^b^
T	0.415 ± 0.06 ^a^	0.443 ± 0.035 ^a^	−0.15 ± 0.052 ^fg^	0.252 ± 0.017 ^bc^
F	0.308 ± 0.045 ^ab^	0.451 ± 0.043 ^a^	−0.304 ± 0.114 ^gh^	0.189 ± 0.017 ^bcd^
SS	0.345 ± 0.13 ^a^	0.531 ± 0.059 ^a^	−0.182 ± 0.074 ^fgh^	−0.063 ± 0.061 ^ef^
S+T	0.034 ± 0.052 ^c^	−0.025 ± 0.128 ^c^	0.121 ± 0.04 ^bcde^	0.054 ± 0.039 ^cde^
S+T+F	0.065 ± 0.087 ^bc^	0.021 ± 0.129 ^c^	−0.34 ± 0.076 ^gh^	0.019 ± 0.048 ^def^
S+T+F+SS	0.374 ± 0.104 ^a^	0.313 ± 0.101 ^ab^	−0.359 ± 0.115 ^h^	−0.158 ± 0.068 ^fgh^
Mg^+2^ levels				
S	−0.028 ± 0.037 ^bcd^	0.15 ± 0.036 ^a^	0.123 ± 0.055 ^a^	0.089 ± 0.071 ^ab^
T	0.058 ± 0.042 ^ab^	−0.063 ± 0.048 ^bcd^	−0.368 ± 0.028 ^efg^	−0.137 ± 0.086 ^cd^
F	0.016 ± 0.044 ^abcd^	−0.041 ± 0.026 ^bcd^	−0.361 ± 0.041 ^efg^	−0.093 ± 0.063 ^bcd^
SS	0.012 ± 0.102 ^abcd^	−0.029 ± 0.035 ^bcd^	−0.181 ± 0.106 ^cde^	−0.052 ± 0.024 ^abc^
S+T	0.038 ± 0.017 ^abc^	−0.094 ± 0.059 ^bcd^	−0.383 ± 0.043 ^fg^	−0.261 ± 0.065 ^def^
S+T+F	−0.033 ± 0.02 ^bcd^	−0.09 ± 0.079 ^bcd^	−0.483 ± 0.042 ^g^	−0.204 ± 0.082 ^cdef^
S+T+F+SS	−0.116 ± 0.054 ^cd^	−0.133 ± 0.025 ^d^	−0.359 ± 0.06 ^efg^	−0.115 ± 0.01 ^cd^
Na^+^/K^+^				
S	2.368 ± 0.15 ^a^	1.776 ± 0.092 ^bc^	3.869 ± 0.158 ^a^	0.534 ± 0.168 ^de^
T	1.784 ± 0.175 ^bc^	0.005 ± 0.052 ^f^	0.9 ± 0.11 ^cd^	0.087 ± 0.252 ^fg^
F	1.137 ± 0.04 ^d^	−1.057 ± 0.039 ^h^	0.54 ± 0.063 ^de^	−0.088 ± 0.014 ^g^
SS	1.458 ± 0.06 ^c^	−0.453 ± 0.184 ^g^	0.505 ± 0.052 ^def^	−0.225 ± 0.112 ^gh^
S+T	2.014 ± 0.103 ^b^	0.478 ± 0.131 ^e^	3.895 ± 0.176 ^a^	2.03 ± 0.205 ^b^
S+T+F	1.505 ± 0.061 ^c^	−0.414 ± 0.044 ^g^	1.085 ± 0.059 ^c^	0.641 ± 0.015 ^de^
S+T+F+SS	1.594 ± 0.109 ^c^	0.015 ± 0.141 ^f^	0.38 ± 0.168 ^ef^	−0.558 ± 0.122 ^h^
Na^+^/Ca^+2^				
S	2.457 ± 0.04 ^a^	1.881 ± 0.048 ^bc^	3.076 ± 0.073 ^a^	0.145 ± 0.203 ^ef^
T	1.414 ± 0.102 ^e^	−0.345 ± 0.055 ^h^	0.499 ± 0.083 ^de^	−0.165 ± 0.241 ^f^
F	0.866 ± 0.051 ^f^	−1.328 ± 0.045 ^i^	0.724 ± 0.186 ^cd^	−0.127 ± 0.022 ^f^
SS	0.683 ± 0.117 ^f^	−1.557 ± 0.046 ^i^	0.433 ± 0.066 ^de^	−0.121 ± 0.051 ^f^
S+T	2.075 ± 0.097 ^bc^	0.352 ± 0.154 ^g^	3.231 ± 0.222 ^a^	1.758 ± 0.197 ^b^
S+T+F	1.697 ± 0.068 ^cd^	0.253 ± 0.097 ^g^	1.134 ± 0.118 ^c^	0.884 ± 0.048 ^cd^
S+T+F+SS	1.437 ± 0.112 ^de^	−0.531 ± 0.133 ^h^	0.875 ± 0.211 ^cd^	0.04 ± 0.142 ^ef^
Na^+^/Mg^+2^				
S	2.188 ± 0.096 ^a^	1.433 ± 0.111 ^d^	3.721 ± 0.153 ^a^	0.356 ± 0.175 ^efg^
T	1.771 ± 0.133 ^c^	0.04 ± 0.062 ^g^	0.717 ± 0.103 ^cde^	0.455 ± 0.224 ^ef^
F	1.214 ± 0.021 ^e^	−0.836 ± 0.011 ^h^	0.514 ± 0.138 ^ef^	0.156 ± 0.069 ^fgh^
SS	1.056 ± 0.036 ^e^	−0.997 ± 0.069 ^h^	0.304 ± 0.045 ^efg^	−0.132 ± 0.046 ^h^
S+T	2.071 ± 0.056 ^ab^	0.289 ± 0.073 ^f^	3.735 ± 0.212 ^a^	2.195 ± 0.193 ^b^
S+T+F	1.852 ± 0.078 ^bc^	0.365 ± 0.109 ^f^	0.999 ± 0.119 ^cd^	1.108 ± 0.078 ^c^
S+T+F+SS	1.944 ± 0.013 ^bc^	−0.085 ± 0.017 ^g^	0.632 ± 0.107 ^de^	−0.002 ± 0.104 ^gh^

Note: Treatment stages are mentioned as S (Seedling); T (Tillering); F (Flowering); SS (Seed setting); S+T (Seeding + Tillering); S+T+F (Seeding + Tillering + Flowering): S+T+F+SS (Seeding + Tillering + Flowering + Seed Setting). Data is presented as mean of fold change ± SE, *n* = 3. All the data are the mean of three replicates ± SE. Different letters indicate significantly different values based on Duncan test across treatments (DMRT, *p* ≤ 0.05), considering the fold change in both Jaya and CSR36 together. The data are presented here is Log2 fold change values with respect to respective controls at respective stages.

**Table 2:** Effects of 50 mM NaCl stress on rice varieties Jaya and CSR36 on Log2 fold change of micro-cation (Fe^+3^, Mn^+2^, Zn^+2^ and Co^+3^) uptake in root and leaf in stage-specific and combined-stage salinity stress.

Micro Cations				
	Root		Shoot	
Treatments	Jaya	CSR36	Jaya	CSR36
Fe^+3^ levels				
S	−0.759 ± 0.405 ^gh^	−0.439 ± 0.079 ^efg^	−0.16 ± 0.074 ^de^	−0.089 ± 0.148 ^cde^
T	0.689 ± 0.104 ^b^	1.594 ± 0.054 ^a^	0.24 ± 0.106 ^abc^	0.558 ± 0.134 ^a^
F	0.616 ± 0.172 ^bc^	1.366 ± 0.094 ^a^	0.165 ± 0.127 ^bcd^	0.322 ± 0.095 ^ab^
SS	−0.584 ± 0.126 ^fgh^	0.256 ± 0.161 ^bcd^	−0.584 ± 0.06 ^f^	−0.081 ± 0.034 ^cde^
S+T	−0.123 ± 0.042 ^de^	0.373 ± 0.026 ^bc^	0.061 ± 0.084 ^bcd^	0.382 ± 0.126 ^ab^
S+T+F	−0.25 ± 0.057 ^ef^	0.218 ± 0.008 ^cd^	−0.083 ± 0.064 ^cde^	0.165 ± 0.044 ^bcd^
S+T+F+SS	−0.95 ± 0.15 ^h^	−0.111 ± 0.018 ^de^	−0.968 ± 0.118 ^g^	−0.284 ± 0.163 ^ef^
Mn^+2^ levels				
S	−1.049 ± 0.356 ^g^	−0.257 ± 0.082 ^de^	−0.641 ± 0.043 ^e^	−0.087 ± 0.062 ^d^
T	0.458 ± 0.196 ^ab^	0.87 ± 0.057 ^a^	−0.006 ± 0.087 ^cd^	1 ± 0.095 ^a^
F	−0.572 ± 0.101 ^ef^	0.474 ± 0.086 ^ab^	−0.604 ± 0.052 ^e^	0.595 ± 0.142 ^b^
SS	−0.262 ± 0.132 ^ef^	0.319 ± 0.078 ^bc^	−0.204 ± 0.075 ^d^	−0.073 ± 0.036 ^d^
S+T	0.264 ± 0.075 ^bc^	0.478 ± 0.02 ^ab^	−0.281 ± 0.048 ^d^	0.55 ± 0.086 ^b^
S+T+F	−1.103 ± 0.063 ^g^	0.069 ± 0.057 ^bcd^	−0.964 ± 0.12 ^f^	0.186 ± 0.125 ^c^
S+T+F+SS	−0.793 ± 0.088 ^fg^	−0.06 ± 0.079 ^cd^	−0.897 ± 0.042 ^f^	−0.227 ± 0.078 ^d^
Zn^+2^ levels				
S	0.513 ± 0.084 ^a^	0.129 ± 0.031 ^bc^	−0.142 ± 0.137 ^ab^	−1.449 ± 0.317 ^e^
T	−0.163 ± 0.074 ^de^	−0.321 ± 0.045 ^defg^	0.121 ± 0.115 ^a^	−0.218 ± 0.11 ^ab^
F	−0.569 ± 0.155 ^g^	0.277 ± 0.089 ^ab^	−0.667 ± 0.08 ^cd^	−0.138 ± 0.073 ^ab^
SS	−0.377 ± 0.064 ^efg^	−0.087 ± 0.06 ^cd^	−0.439 ± 0.099 ^bc^	−0.104 ± 0.128 ^ab^
S+T	−0.276 ± 0.126 ^def^	−0.518 ± 0.096 ^fg^	−0.084 ± 0.042 ^ab^	−0.489 ± 0.074 ^bc^
S+T+F	−0.995 ± 0.054 ^h^	−0.05 ± 0.115 ^cd^	−1.029 ± 0.128 ^d^	−0.354 ± 0.032 ^bc^
S+T+F+SS	−0.903 ± 0.061 ^h^	−0.216 ± 0.067 ^de^	−1.025 ± 0.121 ^d^	−0.305 ± 0.144 ^abc^
Co^+3^ levels				
S	−1.622 ± 0.046 ^f^	−2.21 ± 0.102 ^g^	−1.462 ± 0.498 ^e^	−2.138 ± 0.632 ^e^
T	0.49 ± 0.041 ^b^	−0.062 ± 0.061 ^cd^	0.239 ± 0.08 ^bcd^	0.948 ± 0.287 ^ab^
F	−0.206 ± 0.136 ^de^	0.902 ± 0.089 ^a^	−0.309 ± 0.203 ^cd^	0.751 ± 0.108 ^ab^
SS	−0.159 ± 0.034 ^de^	0.38 ± 0.004 ^b^	−0.258 ± 0.095 ^cd^	0.427 ± 0.077 ^abc^
S+T	1.032 ± 0.011 ^a^	0.01 ± 0 ^cd^	1.129 ± 0.202 ^a^	0.95 ± 0.244 ^ab^
S+T+F	0.122 ± 0.13 ^c^	0.953 ± 0.042 ^a^	−0.227 ± 0.174 ^cd^	0.887 ± 0.175 ^ab^
S+T+F+SS	−0.361 ± 0.019 ^e^	0.119 ± 0.025 ^c^	−0.56 ± 0.085 ^d^	0.219 ± 0.083 ^bcd^

Note: Treatment stages are mentioned as S (Seedling); T (Tillering); F (Flowering); SS (Seed setting); S+T (Seeding + Tillering); S+T+F (Seeding + Tillering + Flowering): S+T+F+SS (Seeding + Tillering + Flowering + Seed Setting). Data are presented as mean of fold change ± SE, *n* = 3. All the data are the mean of three replicates ± SE. Different letters indicate significantly different values based on Duncan test across treatments (DMRT, *p* ≤ 0.05), considering the fold change in both Jaya and CSR36 together.

## Data Availability

The datasets presented in this study can be found in the article/Supplementary Materials.

## Supplementary Materials

The following supporting information can be downloaded at: https://www.mdpi.com/article/10.3390/plants13060778/s1. Figure S1. Effect of salt (50 mM NaCl) on the root (A) and leaf (B) Na, K, Ca and Mg content in Jaya and CSR36 rice varieties subjected to 10 days of stress in pot study; Figure S2. Effect of salt (50 mM NaCl) on the root (A) and leaf (B) Na, K, Ca and Mg content in Jaya and CSR36 rice varieties subjected to 10 days of stress in pot study; Figure S3. Effect of salt (50 mM NaCl) on the root (A) and leaf (B) Fe, Mn, Zn and Co content in Jaya and CSR36 rice varieties subjected to 10 days of stress in pot study; Figure S4. Effect of salt (50 mM NaCl) on the root (A) and leaf (B) chloride, sulphate and phosphate content in Jaya and CSR36 rice varieties subjected to 10 days of stress in pot study; Figure S5. Effect of different levels of salinity (0, 50, 100, 150, 200 mM NaCl) on the root fresh weight (A), root dry weight (B), shoot fresh weight (C) and shoot dry weight (D) in CSR36 and Jaya rice varieties grown in hydroponic media for 7 days followed by 10 days of salt stress; Table S1: Effects of 50 mM NaCl stress on rice varieties Jaya and CSR36 on plant phenotype at stage specific and combined stage salinity stress; Table S2: Effects of 50 mM NaCl stress on rice varieties Jaya and CSR36 on antioxidant enzymes and their substrates at stage specific and combined stage salinity stress; Table S3: Effects of 50 mM NaCl stress on rice varieties Jaya and CSR36 on osmolytes and MDA content at stage specific and combined stage salinity stress.
