# Supplementary material for: Antioxidant Defense and Ionic Homeostasis Govern Stage-Specific Response of Salinity Stress in Contrasting Rice Varieties
Source: Plants (Basel). 2024 Mar 9;13(6):778. doi: 10.3390/plants13060778 (PMC10975454; doi:10.3390/plants13060778)
Supplement: Supplementary file 1 [file plants-13-00778-s001.zip › Supplementary Figures S1-S5.docx]

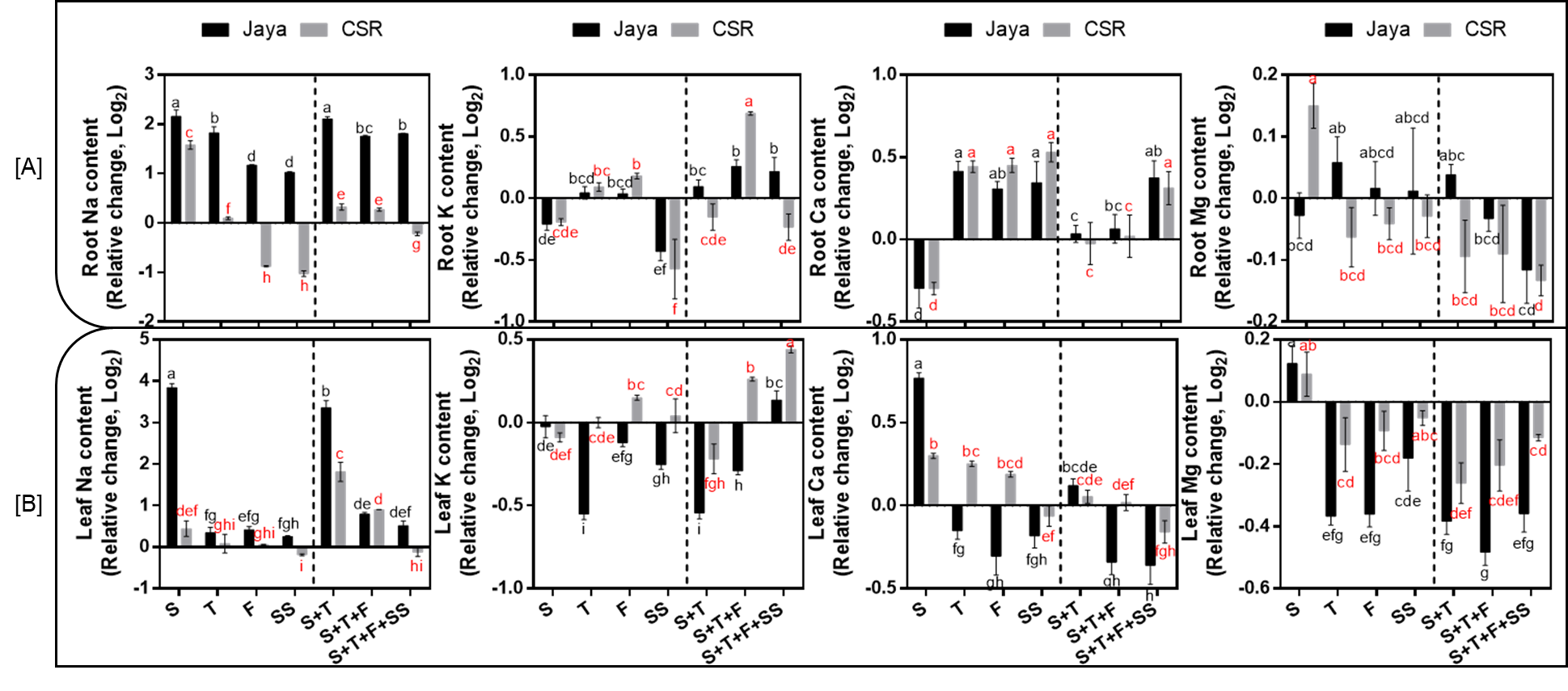


**Supplementary Figure S1.** Effect of salt (50mM NaCl) on the root **(A)** and leaf **(B)** Na, K, Ca and Mg content in Jaya and CSR36 rice varieties subjected to 10 days of stress in pot study at S- (seedling), T- (tillering), F- (flowering), SS- (seed setting) under stage specific and S+T- (seedling + tillering), S+T+F- (seedling + tillering + flowering), S+T+F+SS- (seedling + tillering + flowering + seed setting) stages. The data represented in the form of relative change with respect to respective control and are converted to Log2. Different letters indicate significantly different values across treatments (DMRT, p ≤ 0.05), considering the fold change in both Jaya and CSR36 together.


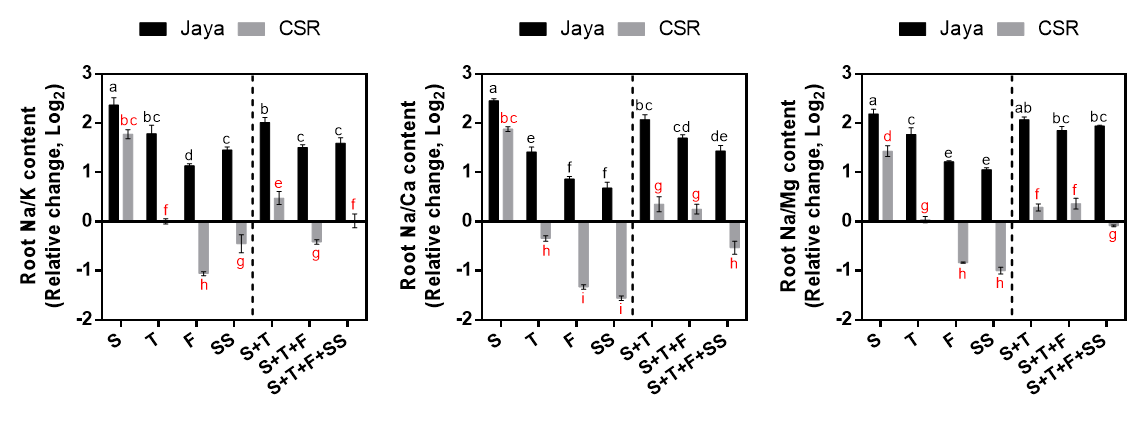

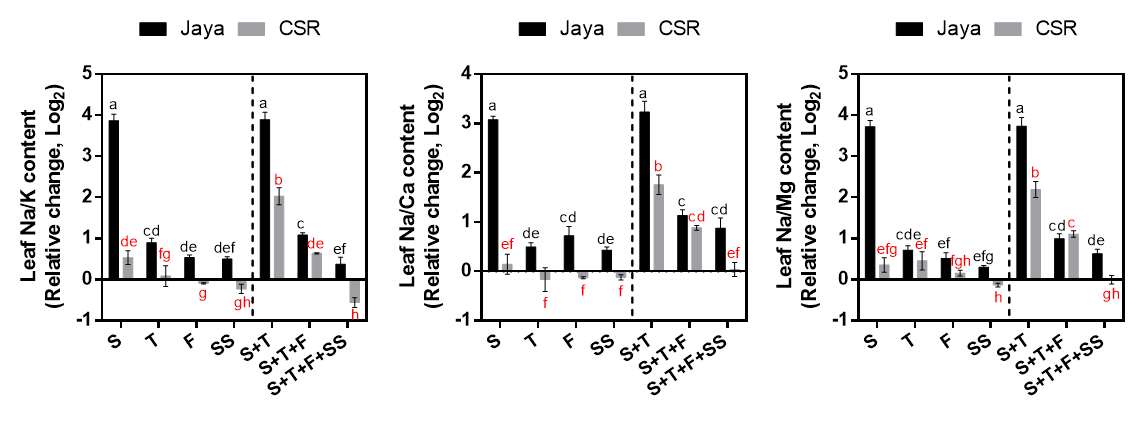


[A]

[B]

**Supplementary Figure S2.** Effect of salt (50mM NaCl) on the root **(A)** and leaf **(B)** Na, K, Ca and Mg content in Jaya and CSR36 rice varieties subjected to 10 days of stress in pot study at S- (seedling), T- (tillering), F- (flowering), SS- (seed setting) under stage specific and S+T- (seedling + tillering), S+T+F- (seedling + tillering + flowering), S+T+F+SS- (seedling + tillering + flowering + seed setting) stages. The data represented in the form of relative change with respect to respective control and are converted to Log2. Different letters indicate significantly different values across treatments (DMRT, p ≤ 0.05), considering the fold change in both Jaya and CSR36 together.


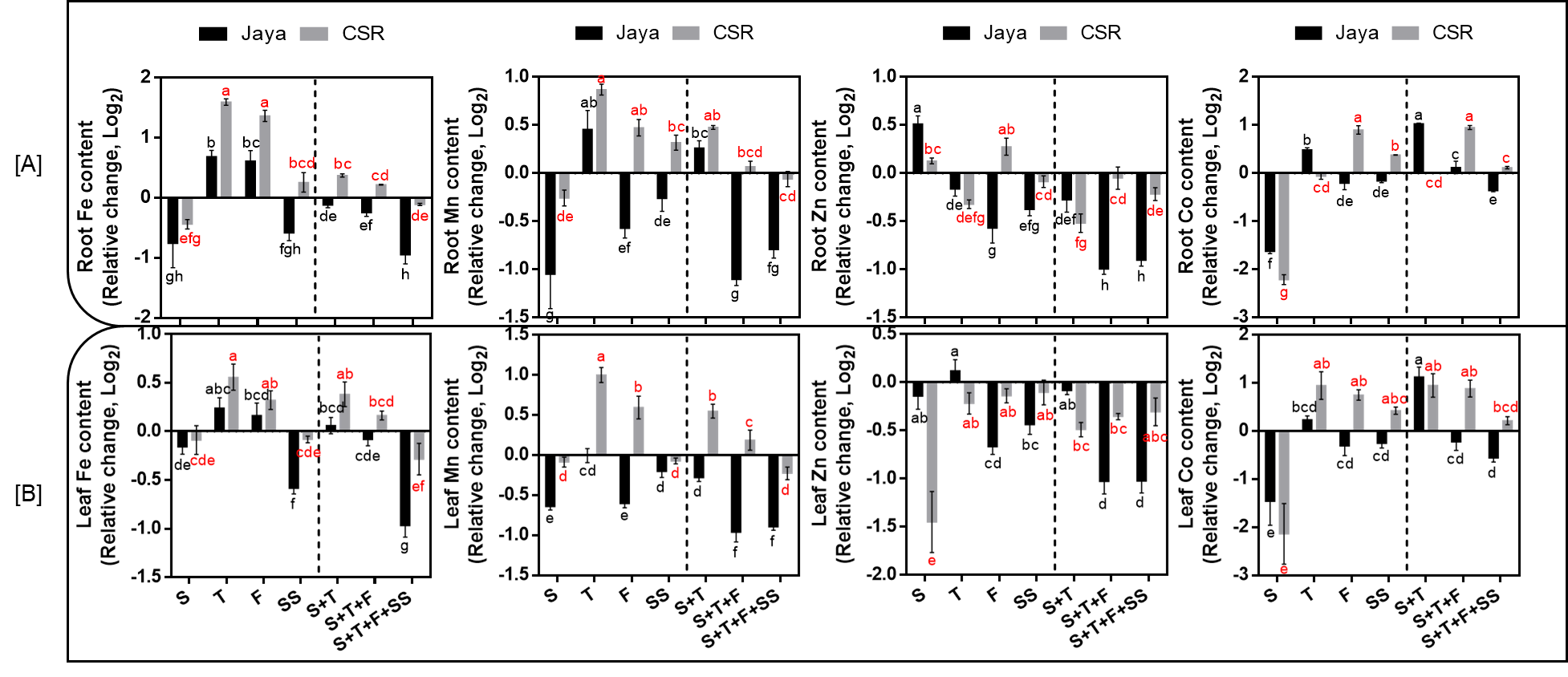


**Supplementary Figure S3.** Effect of salt (50mM NaCl) on the root **(A)** and leaf **(B)** Fe, Mn, Zn and Co content in Jaya and CSR36 rice varieties subjected to 10 days of stress in pot study at S- (seedling), T- (tillering), F- (flowering), SS- (seed setting) under stage specific and S+T- (seedling + tillering), S+T+F- (seedling + tillering + flowering), S+T+F+SS- (seedling + tillering + flowering + seed setting) stages. The data represented in the form of relative change with respect to respective control and are converted to Log2. Different letters indicate significantly different values across treatments (DMRT, p ≤ 0.05), considering the fold change in both Jaya and CSR36 together.


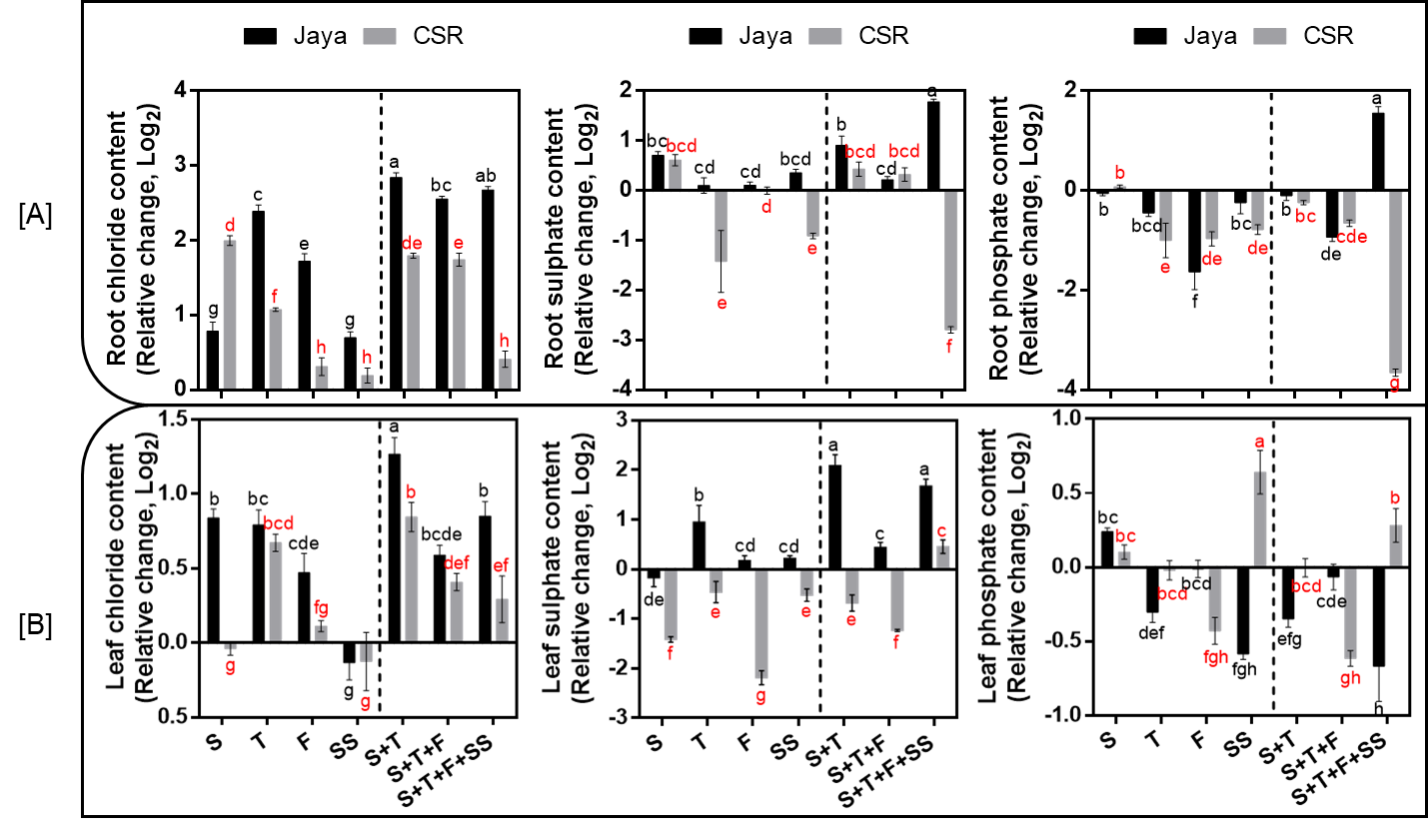


**Supplementary Figure S4.** Effect of salt (50mM NaCl) on the root **(A)** and leaf **(B)** chloride, sulphate and phosphate content in Jaya and CSR36 rice varieties subjected to 10 days of stress in pot study at S- (seedling), T- (tillering), F- (flowering), SS- (seed setting) under stage specific and S+T- (seedling + tillering), S+T+F- (seedling + tillering + flowering), S+T+F+SS- (seedling + tillering + flowering + seed setting) stages. The data represented in the form of relative change with respect to respective control and are converted to Log2. Different letters indicate significantly different values across treatments (DMRT, p ≤ 0.05), considering the fold change in both Jaya and CSR36 together.


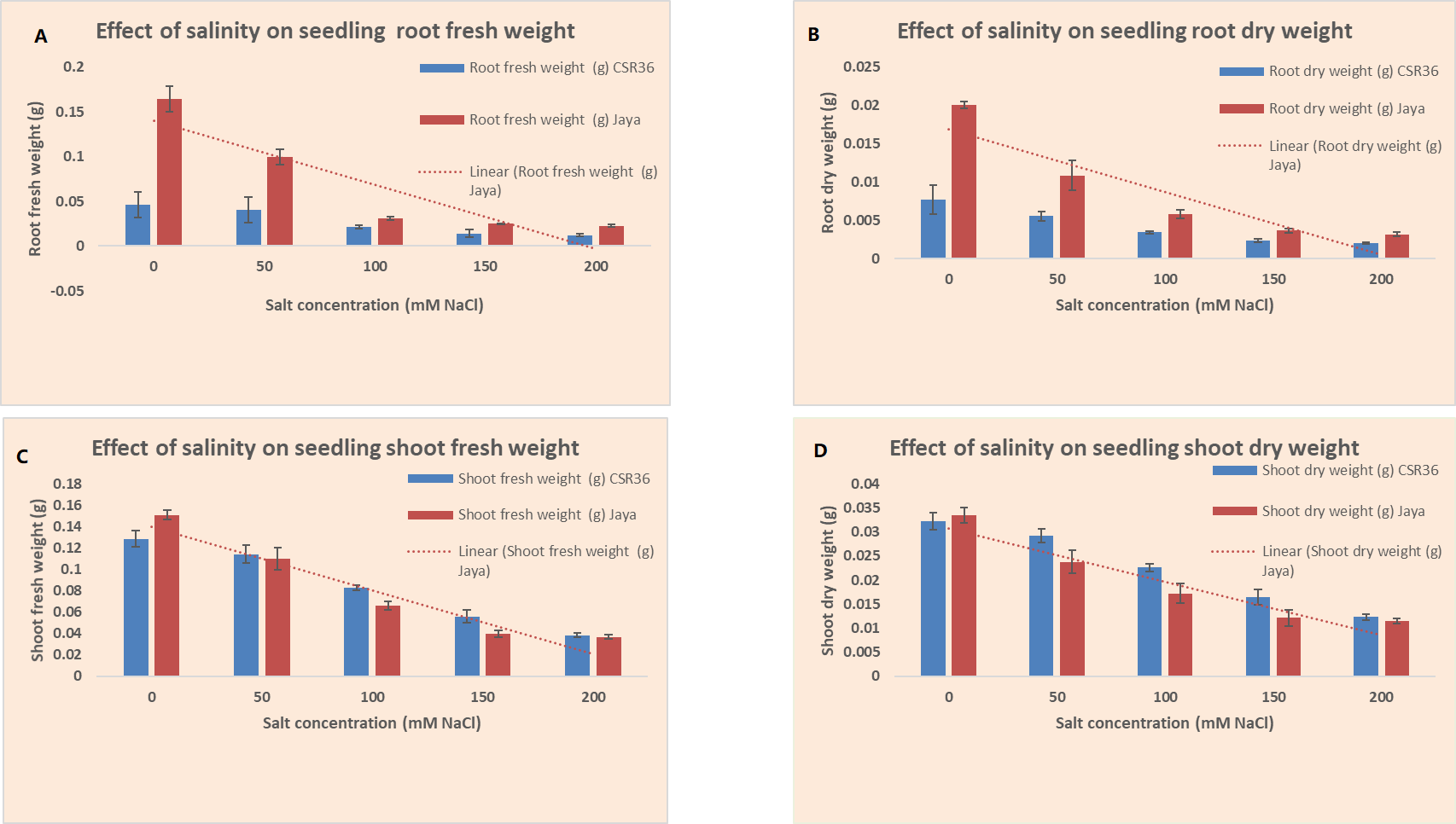


**Supplementary Figure S5.** Effect of different levels of salinity (0, 50, 100, 150, 200mM NaCl) on the root fresh weight **(A)**, root dry weight **(B)**, shoot fresh weight **(C)** and shoot dry weight **(D)** in CSR36 and Jaya rice varieties grown in hydroponic media for 7 days followed by 10 days of salt stress. The data is represented in the form of mean of three replicates.
